# Transcriptome and UPLC-MS/MS reveal mechanisms of amino acid biosynthesis in sweet orange ‘Newhall’ after different rootstocks grafting

**DOI:** 10.3389/fpls.2023.1216826

**Published:** 2023-07-11

**Authors:** Bo Xiong, Qin Li, Junfei Yao, Wei Zheng, Yinghong Ou, Yuanyuan He, Ling Liao, Xun Wang, Honghong Deng, Mingfei Zhang, Guochao Sun, Siya He, Jiaxian He, Xiaoai Zhang, Zhihui Wang

**Affiliations:** College of Horticulture, Sichuan Agricultural University, Chengdu, China

**Keywords:** ‘Newhall’ peel, amino acid, WGCNA, L-Asparagine, L-valine, TFs

## Abstract

Sweet orange ‘Newhall’ (C. sinensis) is a popular fruit in high demand all over the world. Its peel and pulp are rich in a variety of nutrients and are widely used in catering, medicine, food and other industries. Grafting is commonly practiced in citrus production. Different rootstock types directly affect the fruit quality and nutritional flavor of citrus. However, the studies on citrus metabolites by grafting with different rootstocks are very limited, especially for amino acids (AAs). The preliminary test showed that there were significant differences in total amino acid content of two rootstocks (*Poncirus trifoliata* (CT) and *C. junos Siebold ex Tanaka* (CJ)) after grafting, and total amino acid content in the peel was higher than flesh. However, the molecular mechanism affecting amino acid differential accumulation remains unclear. Therefore, this study selected peel as the experimental material to reveal the amino acid components and differential accumulation mechanism of sweet orange ‘Newhall’ grafted with different rootstocks through combined transcriptome and metabolome analysis. Metabolome analysis identified 110 amino acids (AAs) and their derivatives in sweet orange ‘Newhall’ peels, with L-valine being the most abundant. L-asparagine was observed to be affected by both developmental periods and rootstock grafting. Weighted gene co-expression network analysis (WGCNA) combined with Redundancy Analysis (RDA) revealed eight hub structural genes and 41 transcription factors (TFs) that significantly influenced amino acid biosynthesis in sweet orange ‘Newhall’ peels. Our findings further highlight the significance of rootstock selection in enhancing the nutritional value of citrus fruits and might contribute to the development of functional citrus foods and nutritional amino acid supplements.

## Introduction

Sweet orange ‘Newhall’ (C. sinensis), a member of the Rutaceae family’s Citrus genus, originated in USA and is now widely cultivated in various regions of China, including Sichuan, Chongqing, Jiangxi, Hubei, Hunan, and Guangxi. Citrus fruits are highly valued by consumers because of their unique flavor and significant nutritional and therapeutic benefits (Lv et al., 2015). Sweet orange ‘Newhall’ is a kind of citrus with large consumption in the world. The flesh is eaten as fresh food, while the peel is often ignored by people. Citrus peel accounts for about 1/4 of the total fruit area and is the main by-product of citrus processing. Most studies have shown that these unused or “wasted” citrus peels had a lot of biological potential (Marai et al., 2001; Gonzalez-Molina et al., 2010; Akbarian et al., 2016). Citrus peels are used to produce various by-products, including animal feed, fiber, and molasses, as well as to extract bioactives including AAs (Nair et al., 2018). In addition, it is an excellent source of phytochemicals and natural antioxidants that help prevent free radical damage, cancer, and exhibit anti-inflammatory activity (Gonzalez-Molina et al., 2010; Huang and Ho, 2010; Sharma and Vig, 2014). Many studies have demonstrated that citrus peel had higher levels of healthy chemicals than flesh (Barros et al., 2012; Matsuo et al., 2019; Singh et al., 2020; Sharma et al., 2022b). Therefore, the future of citrus peels in the food business is quite promising.

Citrus peel extracts, such as flavonoids, coumarins, terpenes and alkaloids, have been proved to have antibacterial and anti-inflammatory effects, and AAs also have pharmacological effects (Singh et al., 2020; Fei et al., 2022a; Hussain et al., 2022). Due to the lack of in-depth research on citrus AAs, there are few foods or drugs in the market that use AAs in citrus peel as raw materials, so the research on the composition and content of AAs in citrus peel is particularly important. AAs are the basic building blocks of protein with numerous uses in biochemistry, clinical medicine, and food science (Pott et al., 2014; Wang et al., 2017; Itam et al., 2020). Amino acid composition plays an important role in plant growth and development (Mondal et al., 2015). Proteins are made up of 20 AAs and over 1,000 non-essential AAs which are essential in human and animal nutrition (Rodgers, 2014). AAs exist in two forms: free amino acids (FAAs) and bound amino acids (BAAs) (Stabler et al., 2018). BAAs have less of an impact on food taste because they do not degrade immediately after ingestion. Therefore, FAAs became the main amino acid type affecting fruit flavor quality. It is critical to replenish the necessary AAs that the human body cannot generate in order to preserve physiological stability and the progression of complicated enzymatic operations (Keller et al., 2014). L-isoleucine, L-leucine, L-lysine, L-methionine, L-phenylalanine, L-valine, L-threonine, and L-tryptophan are eight essential AAs, which cannot be produced by humans or their synthesis rate is too slow to keep up with metabolic needs (Galili and Amir, 2013; Hasan and Rima, 2021; Lopez and Mohiuddin, 2023).

The proper intake of AAs is essential to improve human health and maintain normal metabolism (Church et al., 2020). For example, glutamate stimulates the synthesis of red blood cells, increases brain cell nourishment, and strengthens learning and memory (Stanimirovic et al., 1999). L-phenylalanine is the raw ingredient used to make epinephrine, thyroxine, and melanin. L-phenylalanine works with L-tyrosine to form essential neurotransmitters and hormones for the body’s glucose and fat metabolism (Tessari et al., 2010). L-tryptophan is another essential amino acid involved in improving sleep quality by increasing the release of serotonin and melatonin and controlling the circadian rhythm of body (Minet-Ringuet et al., 2004). L-threonine has been discovered to have an important function in the creation of mucosal proteins as well as in influencing the gut immune response (Tang et al., 2021). L-lysine has been demonstrated to improve neurological and immune system functions, as well as stimulate protein absorption and utilization (Fini et al., 2001). Several enzymes are involved in amino acid biosynthesis in plants, with each enzyme providing a specific function. For example, the production of aromatic AAs necessary for plant metabolism, such as L-tryptophan and L-phenylalanine, depends on the shikimate pathway (Zulet-Gonzalez et al., 2020; Bergman et al., 2022; Kanaris et al., 2022). 3-deoxy-D-arabino-heptulosonate-7-phosphate synthase (DAHP) is an important enzyme in controlling carbon entering the shikimate pathway (Mir et al., 2015). Furthermore, glutamine synthetase (GS) contains two major isoenzymes, with GS1 thought to be involved in amino acid biosynthesis in photosynthetic as well as non-photosynthetic organisms (Ohashi et al., 2017; Yoneyama and Suzuki, 2019).

Amino acid composition and content in plants varies greatly depending on fertilization manner, soil type, cultivar, fruit maturity and different tissues of the same fruit (Barros et al., 2012; Kim et al., 2022; Lou et al., 2022). Previous study showed that there were significant differences in the ratio of essential AAs to total AAs between green pepper ash and red pepper and different maturity stages (Fei et al., 2022b). Total amino acid content in peel was higher than pulp in quince (Silva et al., 2004). In addition, abiotic stress also influenced amino acid biosynthesis, such as drought, temperature, salinity, darkness, and water potential (Johnová et al., 2016; Zhu, 2016; Hildebrandt, 2018; Batista-Silva et al., 2019). Total amino acid content in FJ (Z. bungeanum cv. ‘Fengjiao’) intolerant to drought stress was higher than HJ (Z. bungeanum cv. ‘Hanjiao’) tolerant to drought stress, which might be a response to drought stress (Hu et al., 2022). Physiological diseases such as oleocellosis also affected amino acid biosynthesis (Xie et al., 2023).

Previous studies have shown that grafting with different rootstocks can affect the differential biosynthesis of metabolites (Dong et al., 2022; Zhang et al., 2022). However, there has been minimal investigation of amino acid components and molecular mechanism of citrus fruits utilizing various rootstock grafting techniques. The previous experiment found that total amino acid content in the peel (72.75 μmol · g^-1^ FW) reached the peak and was higher than flesh (46.69 μmol · g^-1^ FW) during the fruit expansion stage in sweet orange ‘Newhall’ cv. *P. trifoliata*. Therefore, citrus peel was used to study the amino acid biosynthesis in this experiment. We showed hub genes and regulatory mechanisms involved in amino acid biosynthesis pathway using metabolite profiling and transcriptome analysis at various periods of CT and CJ peels in this study. Our findings revealed the amino acid biosynthesis in citrus peels grafted by two distinct rootstocks, laying the groundwork for the development of citrus fruit-related foods.

## Materials and methods

### Plants and sample preparation

**Figure 1:**
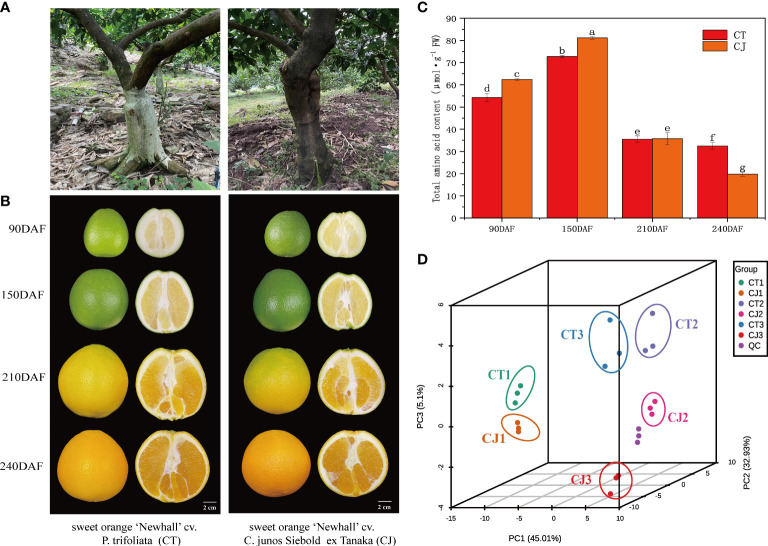
Appearance characteristics of sample material. **(A)** Two distinct appearance characteristics of rootstocks, P. trifoliata and *C. junos Siebold ex Tanaka*. **(B)** Pictures of CT and CJ peels in four different development periods. **(C)** Total amino acid content in CT and CJ peels. Red columns indicating the total amino acid content in CT; orange columns indicating the total amino acid content in CJ. **(D)** PCA analysis of amino acids in different samples.

Two grafting combinations were used as plant material in this study. The first combination was made with sweet orange ‘Newhall’ cv. *P. trifoliata* (CT), while the second combination was made with sweet orange ‘Newhall’ cv. *C. junos Siebold ex Tanaka* (CJ) (**Figure 1A**). Fruit samples of CT and CJ in four different growth stages were collected from 13-year-old trees in Leibo County, Liangshan Prefecture, Sichuan Province, China. The growth stages of CT fruits were identified as CT1 (fruit young period), CT2 (fruit expansion period), CTT (fruit turning period), and CT3 (fruit maturity period) at 90, 150, 210 and 240 days after flowering, respectively (**Figure 1B**). Similarly, CJ fruits were sampled at the same development periods, which were identified as CJ1, CJ2, CJJ, and CJ3, representing 90, 150, 210 and 240 days after flowering. Nine trees were chosen for each stage of CT and CJ, with three trees serving as biological replicates. Twelve fruits from CT and CJ were collected with uniform size and no pests and diseases from five directions (east, south, west, north and middle) of trees in each period, respectively. A total of 96 fruits were collected from two treatments in four periods. The fruit samples were delivered to the lab after being collected, and the pulp and peel were separated. After that, the peels were rapidly precooled in liquid nitrogen before being stored in a -80°C refrigerator for RNA sequencing, amino acid metabolite profiling, and physiological assays. Each experiment had three biological replicates.

### Measurement of total amino acid content

Peels weighing 0.1 g were extracted in 500 μL of a 10% acetic acid solution before being increased to 10 mL with deionized water (Hall and Schonfeldt, 2013). After being ultrasonicated at 25°C, the sample was centrifuged (10 min, 9,000 rpm) (Thermo Sorvall ST16R, USA). The supernatant was transferred using an extra centrifuge tube. Deionized water (1 mL) and sample extract (1 mL) were added in a 15 mL graduated tube. This solution was subsequently treated with 100 L of 0.1% ascorbic acid and three milliliters of ninhydrin. After 15 minutes in a boiling water bath, the supernatant was immediately transferred to an ice bath to cool. The absorbance at 570 nm (Thermo Scientific Multiskan GO, USA) was used as the standard to calculate the leucine equivalents (LEE μmol · g^-1^ FW) of total amino acid content. The tests were repeated three times.

### Metabolite extraction and profiling

Metware Biotechnology Co. Ltd. (Wuhan, China) conducted quantitative and qualitative metabolic experiments. In short, the samples were first frozen and dried after being milled into a powder. A dry sample of 50 mg were immersed in liquid nitrogen for grinding. The extracted sample was then kept refrigerated at 4°C. Centrifugation was performed at 12, 000 rpm at 4°C for 3 min, and the extract was filtered using a membrane with a pore size of 0.22 μm. The extracts were absorbed and filtered before being analyzed using UPLC (Ultra Performance Liquid Chromatography, UPLC) (ExionLC™AD, https://sciex.com.cn/) and MS/MS (Tandem mass spectrometry). The column was Agilent SB-C18 (1.8 μm, 2.1 mm × 100 mm). The mobile phase was made up of ultra-pure water with 0.1% formic acid (liquid A) and acetonitrile with 0.1% formic acid (liquid B). The flow rate was 0.35 mL/min. The following procedure was implemented: The B phase proportion was 5% in zero minute, increased linearly to 95% in zero minute to nine minute, maintained at 95% in nine minute to ten minute, decreased to 5% in ten minute to eleven minute, and balanced at 5% in eleven minute to fourteen minute. The column temperature was 40°C and the sample size was 2 μL. Ion spray voltage was 5,500 V (positive ion mode)/-4,500 V (negative ion mode). The metabolites were characterized according to secondary spectrum information based on metware database. The quantification of metabolites was accomplished by multi-reaction monitoring mode analysis using triple four-bar mass spectrometry.

### MS data analysis

The UPLC-MS/MS detection results were evaluated using AB Sciex 1.6.3 and R software (**Figure S1**). Hierarchical clustering heatmap analysis (HCA) was utilized to evaluate the differential accumulation of AAs in 18 samples. The purpose of the VIP values was to distinguish between the various metabolites. FC ≥ 2 or FC ≤ 0.5, and VIP ≥ 1 were specified as the FC and VIP thresholds, respectively. The different metabolites were annotated using KEGG pathway. A p-value < 0.05 was regarded as significant.

### RNA extraction and transcriptome sequencing

To ensure accuracy and stability, total RNA was isolated with Trizol and concentration and purity were evaluated with a NanoDrop 2000 spectrophotometer (Thermo Scientific, PA, USA). The OD260/280 value of high-quality RNA was determined to be between 1.8 and 2.1. First-strand cDNA and the second strand were synthesized for each RNA sample (1 μg) using Reverse transcription kit (Mei5 Bioservices Co. Ltd, China). Next, the purified double-stranded cDNA was processed to end repair, tail addition, and sequencing adaptor attachment. Eighteen high-quality RNA libraries were constructed and sequenced. High-throughput sequencing was performed using the Illumina Hiseq6000 platform. Trinty was used to splice transcriptome sequencing results to obtain high-quality data. RNA sequencing data were aligned to the *C. sinensis* v3.0 reference genome (http://www.hzau.edu.cn) using HISAT2. Fragments per kilobase of transcript per million fragments mapped (FPKM) was calculated using StringTie. When genes met the criteria for FDR < 0.05 and |log_2_FC| ≥ 1, they were considered differentially expressed genes (DEGs). The R software package was used to analyze DEGs. Raw transcriptome data has been uploaded to the NCBI Sequence Read Archive (BioProject: PRJNA946264).

### Co-expression network and bioinformatic analysis

Co-expression network analysis was performed on a similarity threshold of 0.8 using the R package. The trait file included the contents of differentially accumulated AAs. Modules with high connection to AAs were identified and produced in various colors using the Dynamic Hybrid Tree Cut algorithm. The brown module was used to construct the co-expression network and visualize it using Cytoscape software (Version 3.9.1). The following WGCNA parameters were set: FPKM ≥ 1; the variation of FPKM cv ≥ 0.5; co-expression network: unsigned; power: 18; lower module limit: 50, and combined module minimum height: 0.25.

### Quantitative real-time polymerase chain reaction (qRT-PCR)

Specific primers for qRT-PCR were designed using Primer Premier 5.0 and were provided in **Table S1**. qRT-PCR was performed with SYBR Green Premix Pro Taq HS Kit (Mei5 Bioservices Co. Ltd, China) at 12.5 μL volume. The setting program in a CFX96 Real-Time PCR System (Hercules, CA, USA) was as follows: 95°C for 2 min, 39 cycles at 95°C for 5 s, 45-55°C for 40 s. An internal control was made using actin (**Table S1**). Three duplicates of each sample were performed out with the 2^−△△Ct^ method to calculate cycle threshold values.

### Statistical analysis

Duncan’s test in one-way analysis of variance was used to determine significant differences (P < 0.05). Pearson’s correlation analysis was calculated using SPSS Statistics 2022 (IBM Inc., Chicago, IL, USA). The hierarchical cluster heatmap analysis (HCA) was plotted using the heatmap2 function. Principal component analysis (PCA), orthogonal projections to latent structures discriminant analysis (OPLS-DA), and correlation analysis and RDA were performed using the Metware Cloud (https://cloud.metware.cn). Column charts were illustrated using Origin 2023 (Originla, MA, United States).

## Results

### Accumulation of amino acid metabolites of CT and CJ peels

During the ripening process, fruit gradually enlarge (**Figure 1B**) and total amino acid content had significant difference between CT and CJ. Total amino acid content was significantly higher in CJ than in CT during fruit young period and fruit expansion period, while during fruit maturity period, total amino acid content in CT was higher than that in CJ (**Figure 1C**). Therefore, three developmental stages (90DAF, 150DAF and 240DAF) were selected for the following experiment. UPLC-MS/MS analysis was performed on 18 samples from three stages to investigate the differential metabolites (**Figure S2**). PCA showed that the contribution rate of PC1, PC2 and PC3 was 83.04%, of which the contribution of PC1 was 45.01%, PC2 was 32.93%, and PC3 was 5.1% (**Figure 1D**). OPLS-DA analysis also uncovered significant differences between samples and different developmental stages (**Figure S3**). PCA and OPLS-DA both demonstrated high sample repeatability and could be utilized for further analysis.

### Identification of differential accumulation amino acids (DAAs)

**Figure 2:**
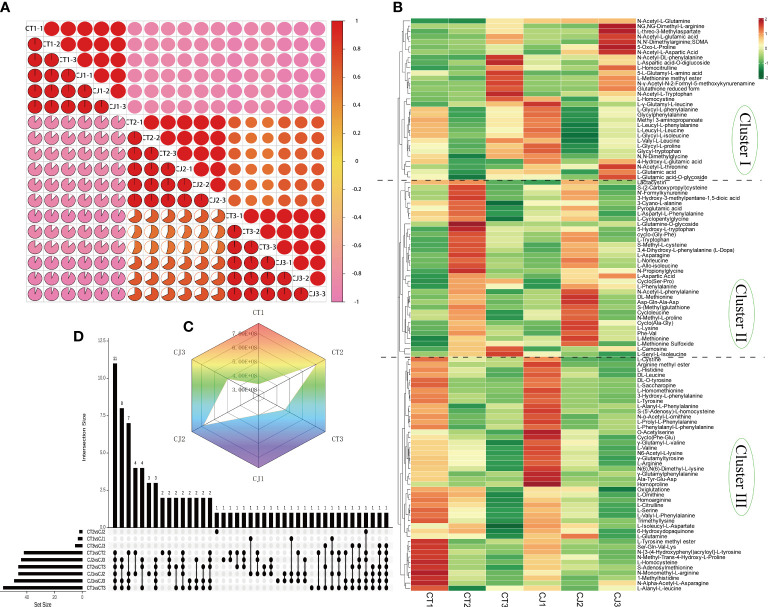
Screening for differentially accumulated AAs in CT and CJ peels. **(A)** Intragroup correlation of 18 samples at different developmental stages. **(B)** Hierarchical clustering heatmap analysis (HCA) indicating the abundance of 110 AAs in CT and CJ peels. Red and green representing the high and low amino acid abundance, respectively. **(C)** Radar chart of Log_2_FC value of the relative level of total amino acid content in each group. **(D)** The column chart showing the numbers of each group.

Intragroup correlation analysis showed significant correlation in biological replicates (**Figure 2A**), indicating that the metabolite data were repeatable and reliable. Metabolite profiling analysis identified 110 AAs and their derivatives, including 5 essential AAs (**Table S2**), which was uncovered to have a large amount of AAs in citrus for the first time. HCA classified 110 AAs into three groups (**Figure 2B**). Cluster I consisted of 31 AAs showing lower content in CT and CJ peels during fruit expansion stage, including L-homocystine. Cluster II comprised 34 AAs, including L-tryptophan, L-asparagine, L-phenylalanine, L-lysine, and L-methionine. Cluster III included 45 AAs, with a greater concentration in fruit young period. The overall trend showed that amino acid levels increased in both CT and CJ peels from fruit young period to fruit expansion period and then decreased from fruit expansion period to fruit maturity period, which was consistent with total amino acid content (**Figure 2C**). These findings revealed significant differences in amino acid content in CT and CJ peels. Among these 110 AAs, 5 were essential AAs with L-valine being the most abundant in 90 DAF, but L-Phenylalanine was the highest in 210 DAF and 240 DAF (**Figure S3**).

**Figure 3:**
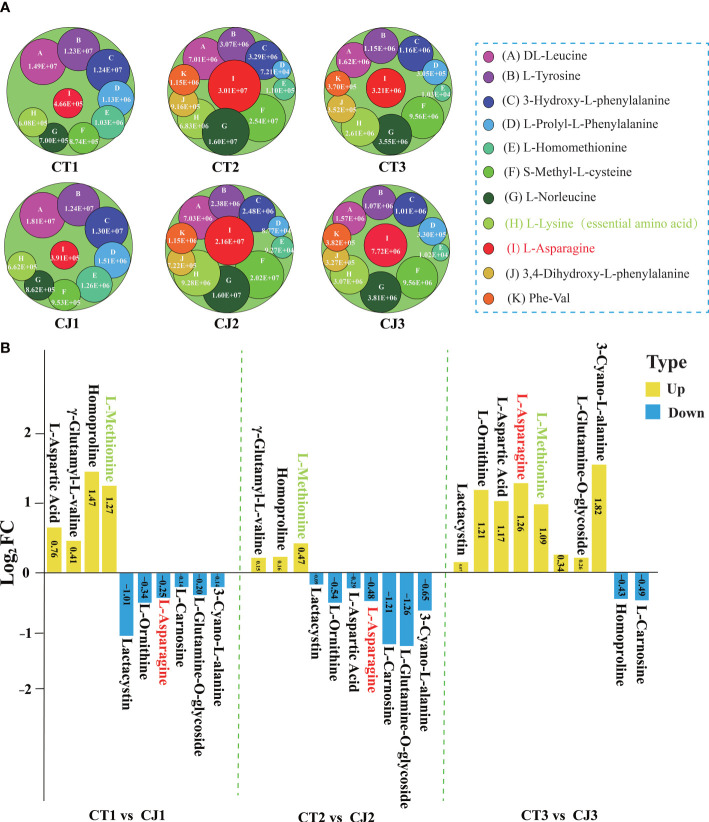
Differential analysis of 20 AAs affected by different developmental stages and different rootstocks grafting in CT and CJ peels. **(A)** 11 AAs affected by different developmental periods content variation. A circle represented one amino acid, and A-K represented each of the 11 AAs. The larger the circle, the higher the relative amino acid content. **(B)** Log_2_FC value of 10 AAs influenced by different rootstocks grafting in CT1 vs CJ1, CT2 vs CJ2, and CT3 vs CJ3, respectively. The yellow columns indicated that the relative amino acid content was up-regulated and the blue columns represented down-regulated in CT vs CJ. The red text in **(A, B)** represented amino acid affected by both developmental stages and rootstock grafting. The green text represented essential amino acid.

The column chart analysis revealed the presence of 42, 57, and 46 DAAs in CT1 vs CT2, CT1 vs CT3, and CT2 vs CT3, respectively. Similarly, 46, 49, and 44 DAAs were identified in CJ1 vs CJ2, CJ1 vs CJ3, and CJ2 vs CJ3, respectively. Interestingly, we discovered that 11 DAAs were common and their contents were significantly different in the six comparison groups (CT1 vs CT2, CT2 vs CT3, CT1 vs CT3, CJ1 vs CJ2, CJ2 vs CJ3 and CJ1 vs CJ3), indicating that the biosynthesis of these AAs was influenced by developmental stage (**Figure 2D**; **Table S2**). We also found an essential amino acid (L-lysine) in these DAAs (**Table S2**). In order to further investigate their changes in different developmental stages, we analyzed the content of these AAs in CT1, CT2, CT3, CJ1, CJ2 and CJ3, respectively (**Figure 3A**). The six AAs content (S-Methyl-L-cysteine, L-Norleucine, L-Lysine, L-Asparagine, 3,4-Dihydroxy-L-phenylalanine and Phe-Val) peaked in August, indicating that these AAs contributed to the higher total amino acid content in the fruit expansion stage. In addition, 3,4-Dihydroxy-L-phenylalanine and Phe-Val were not detected in June, and their content reached a peak in August, until the content decreased in the fruit ripening stage.

Ten AAs were affected by grafting with different rootstocks in 110 AAs, including one essential amino acid (L-Methionine) (**Figure 2D**; **Table S2**). We compared CT and CJ in pairs over three periods to identify DAAs affected by different rootstocks grafting (**Figure 3B**). Four AAs were upregulated and 6 AAs were downregulated in CT1 vs CJ1. Compared with CJ2, 3 AAs were upregulated and 7 AAs downregulated, but 8 AAs were upregulated and 2 AAs downregulated in CT3 vs CJ3. Among them the content of essential amino acid (L-Methionine) was upregulated in the three periods, and L-Asparagine upregulated in mature period, only downregulated in the other two stages. The results showed that the effects of rootstock grafting on different amino acid were significantly different. Furthermore, the presence of L-asparagine in 21 AAs revealed that it was affected not only by developmental periods but also by rootstock grafting.

### Transcriptome overview

**Figure 4:**
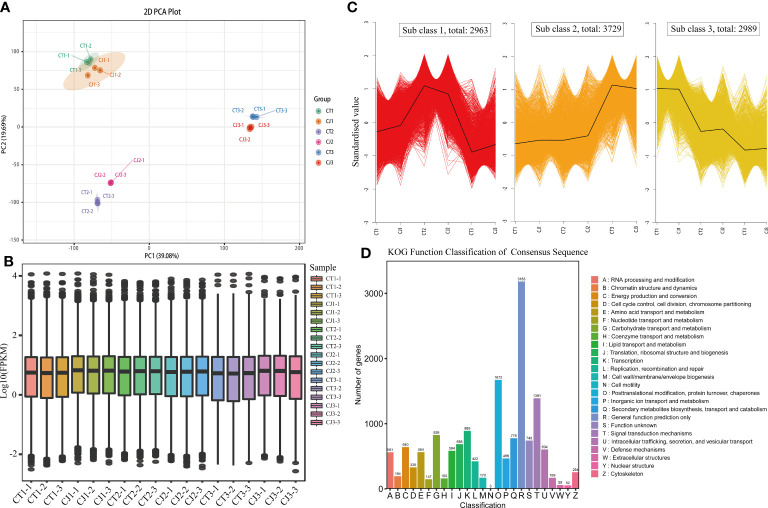
Transcriptome sequencing and functional analysis of DEGs in CT and CJ peels. **(A)** PCA analysis of CT and CJ peels transcriptomes. **(B)** The boxplot of FPKM. **(C)** Kmeans of DEGs. **(D)** KOG function classification of consensus sequence.

The expression levels of genes in CT and CJ peels were analyzed using PCA and boxplot analysis (**Figures 4A, B**). PCA analysis revealed that a contribution rate of 39.08% and 19.69% for PC1 and PC2, respectively (**Figure 4A**). The gene expression trends were comparable in CT1 and CJ1, but there were significant differences between fruit expansion and fruit maturity. Boxplot analysis of FPKM showed significant differences in gene expression characteristics in CT and CJ samples, as well as differences in different developmental stages (**Figure 4B**). The combination of PCA and boxplot analysis demonstrated good biorepeatability, indicating their suitability for subsequent analysis. A total of 9,681 DEGs were detected during three periods in CT and CJ peels (**Figure 4C**; **Table S3**), and these genes were classified into three subclasses: subclass 1 (2,963 DEGs), subclass 2 (3,729 DEGs), and subclass 3 (2,989 DEGs). The DEGs pattern was consistent with total amino acid content in subclass 2. Furthermore, the KOG functional classification of consensus sequence results revealed that 564 genes were found to be involved in amino acid transport and metabolism (**Figure 4D**).

**Figure 5:**
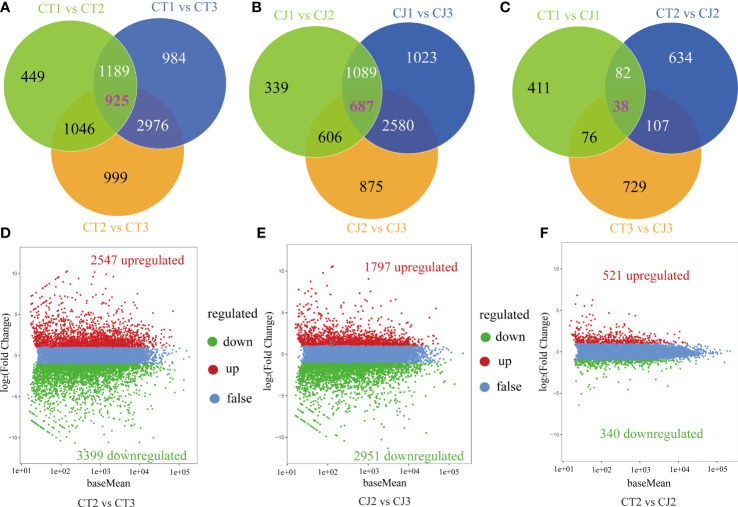
Analysis of DEGs enrichment pathways and expression patterns. **(A-C)** Venn diagrams of DEGs in CT and CJ peels. **(D-F)** Volcano plot of DEGs in CT2 vs CT3, CJ2 vs CJ3 and CT2 vs CJ2, respectively. Red dots indicating the upregulated genes, green dots indicating the downregulated genes, and blue dots indicating the genes with no change significantly.

To further investigate the changes in these DEGs, we performed venn diagrams and volcano plots (**Figure 5**). In CT, 449, 984, and 999 unigenes (FPKM > 1) were shared in CT1 vs CT2, CT1 vs CT3 and CT2 vs CT3, respectively, and 925 genes were identified across the three periods in CT (**Figure 5A**). In CJ, 339, 1,023, and 875 unigenes were present in CJ1 vs CJ2, CJ1 vs CJ3 and CJ2 vs CJ3, respectively. And 698 unigenes were shared by three different developmental stages in CJ (**Figure 5B**). In addition, 411, 634, and 729 unigenes were discovered in CT1 vs CJ1, CT2 vs CJ2 and CT3 vs CJ3, respectively (**Figure 5C**). A total of 5,946 DEGs were identified in CT2 vs CT3, with 2,547 genes upregulated and 3,399 genes downregulated (**Figure 5D**). In CJ2 vs CJ3, 4,748 DEGs were discovered (**Figure 5E**). We found 861 DEGs in CT2 vs CJ2, of which 521 was upregulated and 340 downregulated (**Figure 5F**).

**Figure 6:**
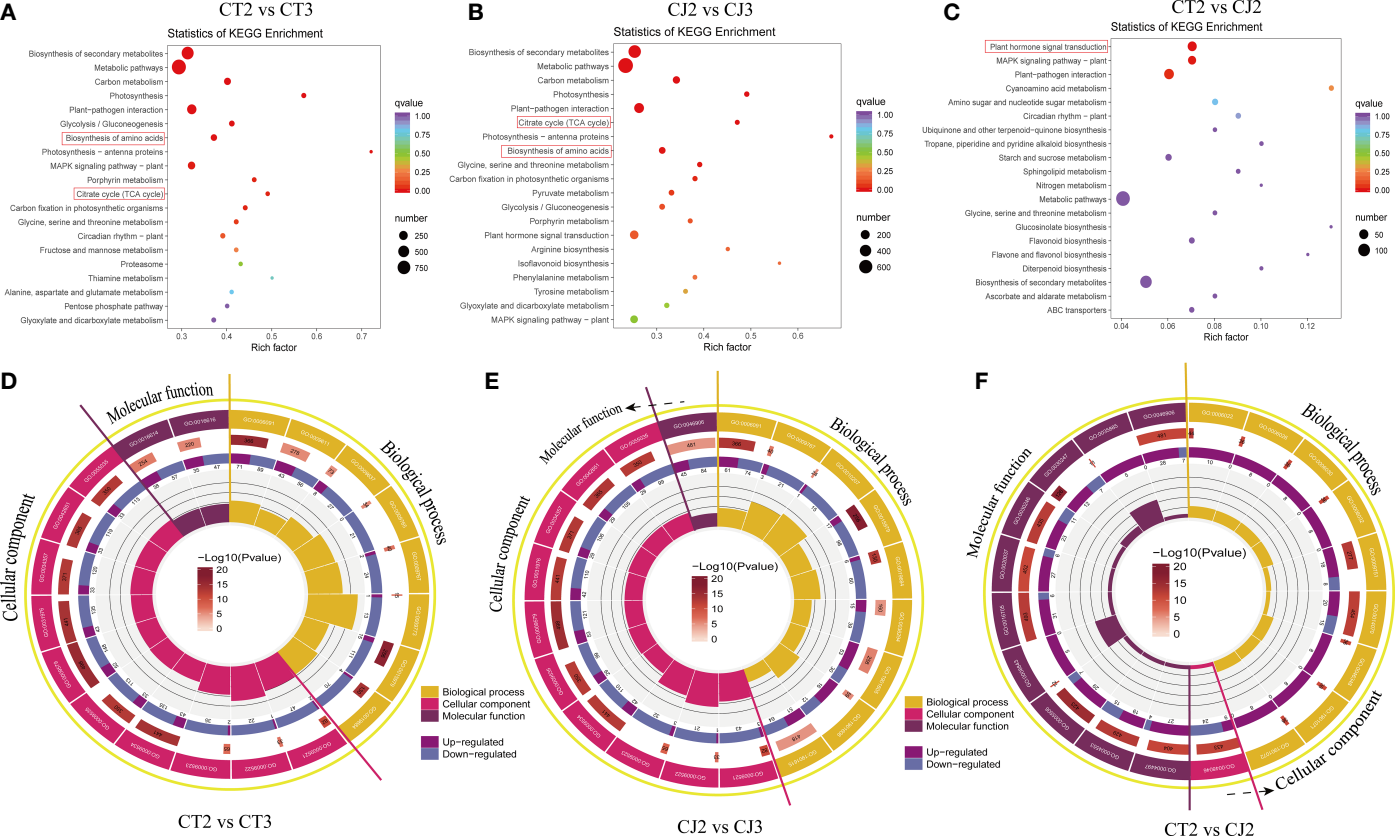
Analysis of DEGs enrichment pathways and expression patterns. **(A-C)** Kyoto encyclopedia of genes and genomes (KEGG) enrichment bubble diagrams of DEGs in CT2 vs CT3, CJ2 vs CJ3 and CT2 vs CJ2, respectively. Bubble size indicating the number of DEGs enriched in this KEGG pathway; bubble color indicating the *P*-values. **(D-F)** GO enrichment circle diagram of DEGs in CT2 vs CT3, CJ2 vs CJ3 and CT2 vs CJ2, respectively. The outside lap represents the top 20 GO terms; the mid lap represents the numbers of all genes in GO terms and *P*-values for gene enrichment for the specified GO term; and the inner lap represents the numbers of DEGs. The ladder column in the center represents the Rich factor of DEGs for each GO term.

In this study, KEGG and GO analysis were used to identify possible DEGs in citrus peels. KEGG bubble map revealed that multiple pathways related to amino acid biosynthesis were significantly enriched in CT and CJ (**Figures 6A–C**). Metabolic pathways, carbon metabolism, glycolysis or gluconeogenesis, photosynthesis, biosynthesis of amino acids and citrate cycle (TCA cycle) were significantly enriched in CT2 vs CT3 and CJ2 vs CJ3. There were three classes of the top 20 GO terms, including generation of precursor metabolites and energy (GO:0006091) in biological process (BP), thylakoid (GO:0009579) in cellular component (CC) in CT2 vs CT3, and oxidoreductase activity (GO:0016614) in molecular function (MF) (**Figure 6D**). The top 20 GO terms in CJ2 vs CJ3 included generation of precursor metabolites and energy (GO:0006091) in BP, thylakoid (GO:0009579) and photosystem (GO:0009521) in CC, and tetrapyrrole binding (GO:0046906) in MF (**Figure 6E**). In addition, polysaccharide binding (GO:0030247) was enriched in MF and response to organic cyclic compound (GO:0014070) in BP between CT2 and CJ2 (**Figure 6F**).

### Identification of amino acid biosynthesis pathway

**Figure 7:**
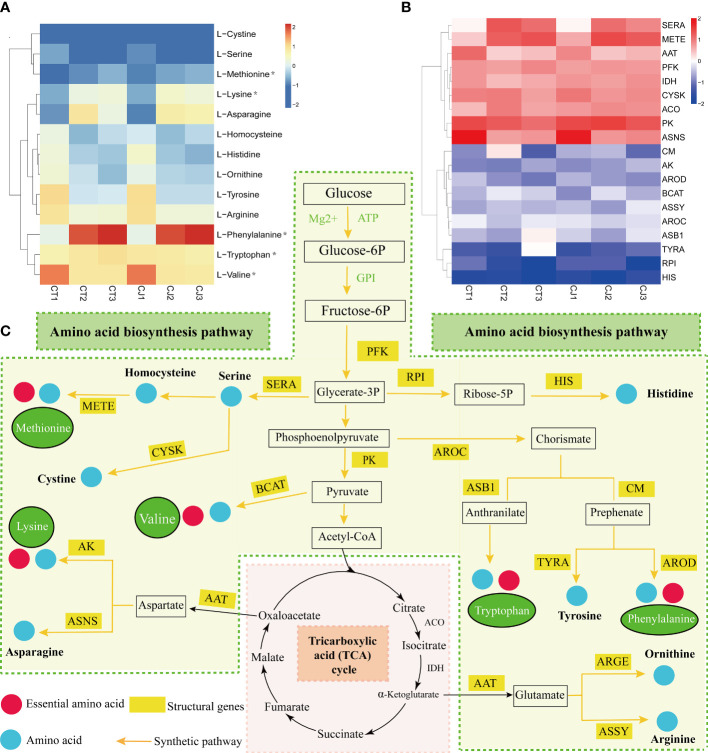
Amino acid synthesis pathway in CT and CJ peels. **(A)** Content distribution of main amino acids in CT and CJ during different maturity stages. Asterisks represent essential amino acids. **(B)** Expression patterns of genes related to amino acid synthesis in different maturity stages of CT and CJ. **(C)** Relationship between structural genes and metabolite synthesis in the amino acid biosynthesis pathway.

We first mapped the amino acid biosynthesis pathway, screened 17 genes related to amino acid synthesis, and analyzed the correlation between the amino acid content of CT and CJ peels and structural genes (**Figures 7A–C**). Heatmap of gene expression levels showed different expression patterns of structural genes in CT and CJ peels (**Figure 7B**). L-homocysteine, L-histidine, L-ornithine, L-tyrosine, L-arginine, and L-valine content decreased in both CT and CJ peels. Correspondingly, the expression levels of D-3-phosphoglycerate dehydrogenase ribose-5-phosphate isomerase (*RPI*), cysteine synthase (*CYSK*), aspartate aminotransferase (*AAT*), pyruvate kinase (*PK*) and asparagine synthetase (*ASNS*) also decreased. L-histidine content decreased with decreased expression of *RPI* in CT1 and CT2, and CJ2 and CJ3. Similarly, L-histidine content decreased when the expression level of histidinol dehydrogenase (*HIS*) decreased in CJ1 and CJ2. L-valine content decreased with the increase of pyruvate kinase (*PK*) and branched-chain-amino-acid aminotransferase (*BCAT*) expression. In contrast, L-phenylalanine accumulated when the expression level of chorismate mutase (*CM*) increased, and L-tyrosine decreased with the expression of prephenate dehydrogenase (*TYRA*). When the expression level of 5-methyltetrahydrofolate-homocysteine (*METE*) increased, L-methionine content accumulated. L-cystine and L-serine content decreased with low expression level of cysteine synthase (*CYSK*). In young fruit stage and fruit expansion stage, the L-lysine increased with higher gene expression level of *aspartokinase* (*AK*), while L-ornithine and L-arginine decreased with lower gene expression level of *AAT*. The amino acid biosynthesis pathway clearly revealed the link between amino acid content and structural genes, which provided the groundwork for further research of amino acid biosynthesis in CT and CJ peels (**Figure 7C**). With a few exceptions, the majority of amino acid content corresponded to the expression pattern of structural genes.

### Correlation analysis and RDA between amino acid content and expression of structural genes

**Figure 8:**
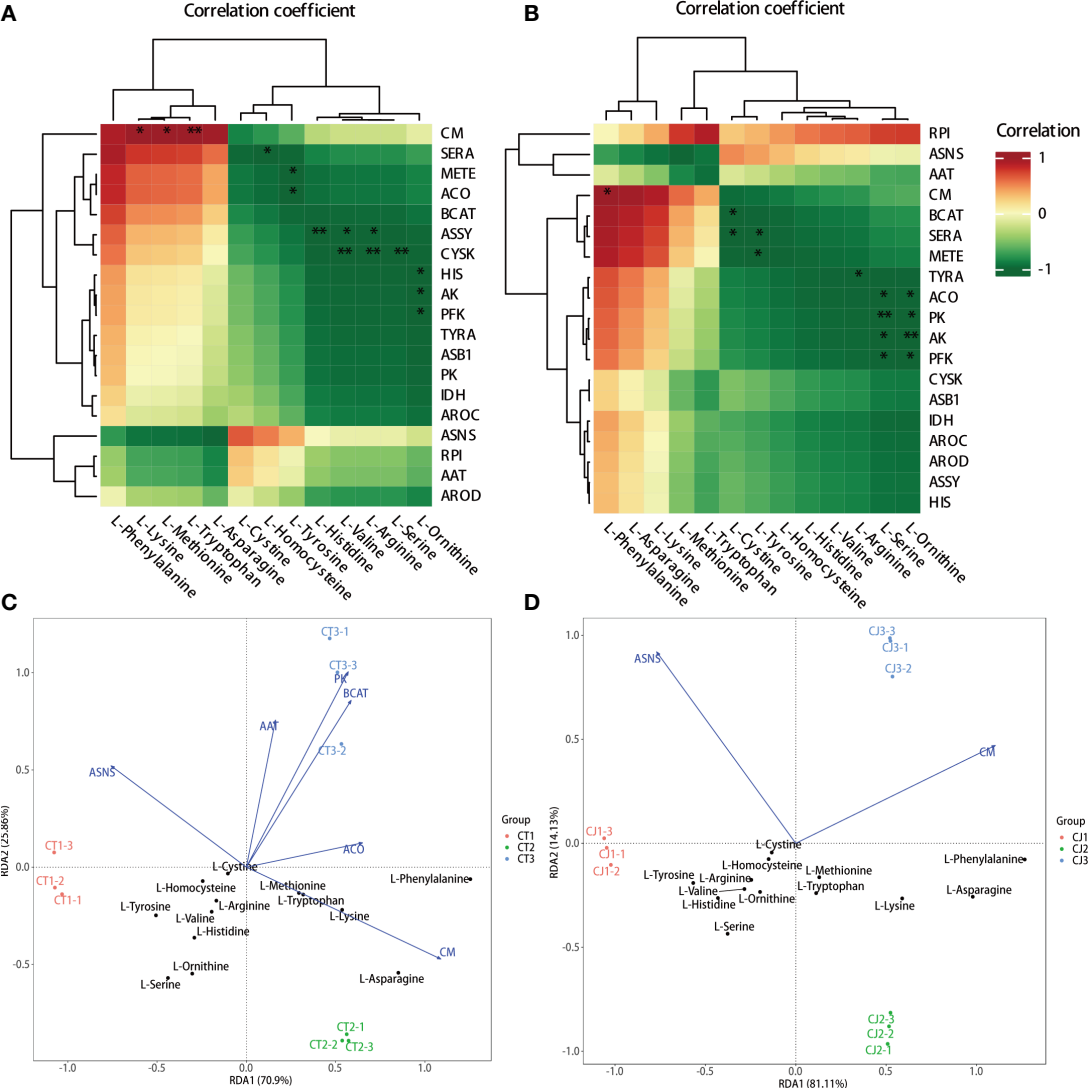
Correlation analysis and redundancy analysis (RDA) of amino acids and synthetic genes in amino acid biosynthesis pathway. **(A, B)** Intergroup analysis of amino acids and synthetic genes in CT and CJ, respectively. **(C, D)** RDA of amino acids and synthetic genes in CT and CJ, respectively. * represents p<0.05; ** represents p<0.01.

The hub genes involved in amino acid biosynthesis were studied by intergroup correlation analysis of amino acid content and structural genes in citrus peels (**Figure 8**). The results showed that L-lysine, L-methionine, and L-tryptophan were positively correlated with *CM* in CT, but L-phenylalanine was positively correlated with the majority of the gene expression. In contrast, L-homocysteine, L-tyrosine, L-histidine, L-valine, L-arginine, L-serine, and L-ornithine negatively correlated with the expression of *SERA*, *METE*, *ACO*, *ASSY*, *CYSK*, *HIS*, *AK*, and *PFK*, respectively (**Figure 8A**). *RPI* gene expression level was associated with the most AAs in CJ, and L-phenylalanine was significantly correlated with the expression of *CM* (**Figure 8B**). *BCAT*, *SERA*, *METE*, *TYRA*, *ACO*, *PK*, *AK*, and *PFK* gene expression were shown to negatively correlate with L-cystine, L-tyrosine, L-arginine, L-serine, and L-ornithine. Notably, *RPI* might play a crucial role in amino acid biosynthesis in CJ rather than CT because of positively correlated with the most AAs content in the biosynthesis pathway. As a result, the genes with a positive connection could be important for amino acid biosynthesis.

In this study, RDA analysis was performed using 13 AAs content and the expression levels of 19 structural genes related to amino acid biosynthesis pathway (**Figures 8C, D**). RDA results revealed that the expression levels of structural genes accounted for 96.76% and 95.24% of the contribution rate in CT and CJ, respectively, providing reliable and reproducible results. Furthermore, the results showed a distinct difference between samples, indicating that different rootstock grafting and developmental stages significantly influenced amino acid biosynthesis. The expression levels of *CM* and L-asparagine, L-lysine, L-methionine, L-tryptophan, and L-phenylalanine were significantly correlated in CT. However, there was no significant correlation between the gene expression level and the aforementioned AA content in CJ. Overall, our results indicate that *CM*, *RPI*, *BCAT*, *SERA*, *METE*, and *ACO* play essential roles in amino acid biosynthesis in sweet orange ‘Newhall’ peels, highlighting their importance in amino acid accumulation during citrus development. Furthermore, our findings suggest that different rootstock grafting and developmental stages have a significant impact on amino acid biosynthesis.

### Co-expression network analysis of DEGs

Co-expression network was performed in this study to identify the hub genes of CT and CJ peels combined with metabolic profiling. The genes enriched in each module showed a comparable pattern of gene expression and were represented by a distinct hue (**Figures S5**, **S6**). Correlation analysis of distinct modules and features was performed to search for the gene clusters associated to amino acid biosynthesis. More than 3,000 genes were uncovered in brown, blue, and turquoise modules, while tan and salmon modules had only 65 and 50 enriched genes, respectively. The brown module showed a relatively high correlation coefficient, which was significantly correlated with L-serine (R = 0.94, P = 0.0053), L-cystine (R = 0.93, P < 0.05), L-homocysteine (R = 0.96, P = 0.0024), L-histidine (R = 0.95, P = 0.0037), L-tyrosine (R = 0.98, P < 0.05), L-valine (R = 0.9, P = 0.014), L-ornithine (R = 0.81, P = 0.051), and L-arginine (R = 0.91, P = 0.012) (**Figure S6B**). A heatmap in conjunction with a bar chart was used to visualize how the gene expression pattern varied in the brown module (**Figure S6C**). Notably, most genes in the brown module had higher expression levels in the young fruit stage compared to the fruit expansion stage before decreasing in the fruit expansion stage vs the fruit maturity stage. This trend was consistent with the varying total amino acid content in each period. Therefore, the brown module was selected to further screen for hub genes.

**Figure 9:**
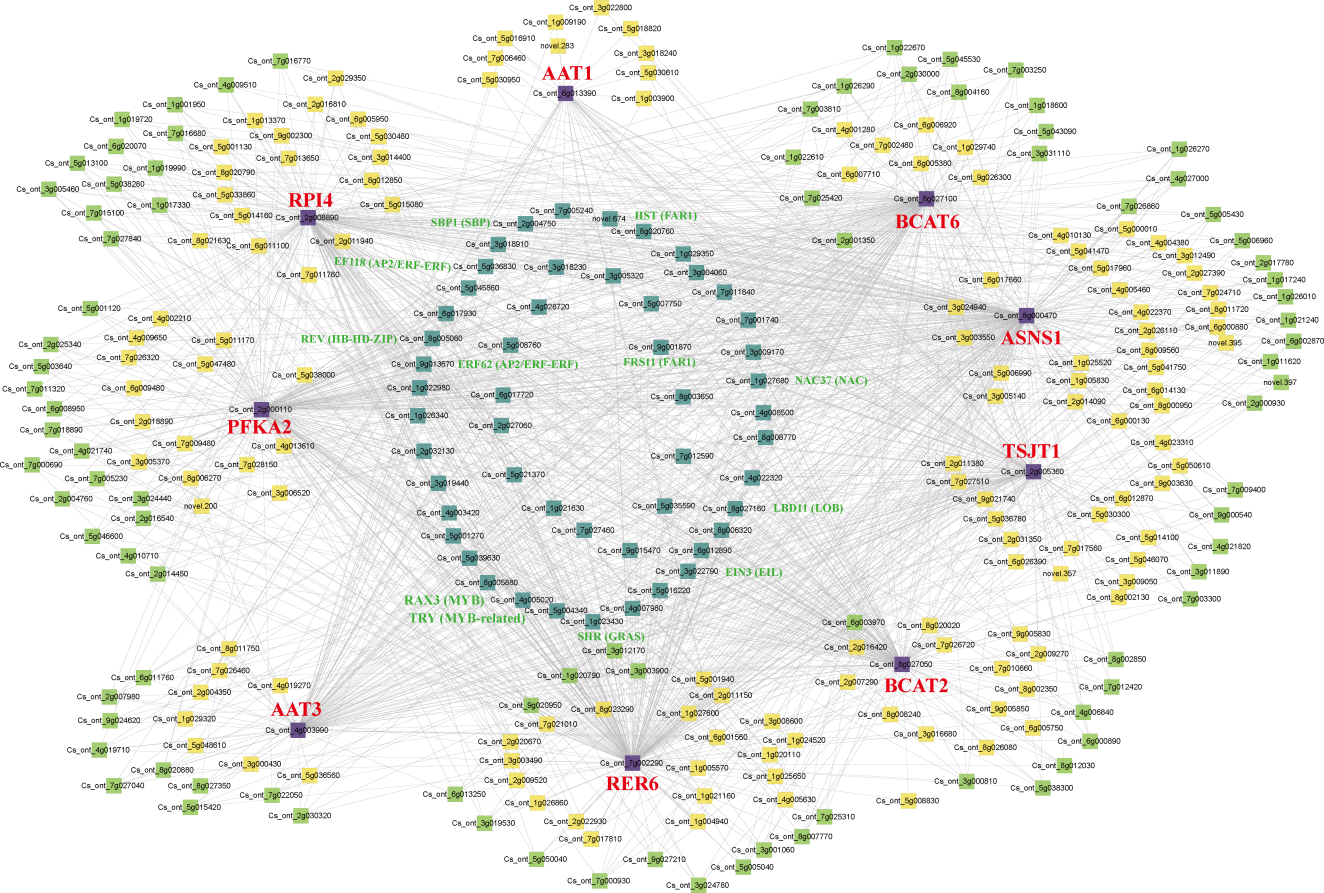
Co-expression network of structural genes with TFs in the brown module. The different colors represent the degree of connectivity. The purple represents structural genes. The cyan represents a high correlation with at least seven structural genes. The green shows associations with five or six structural genes. The yellow is associated with less than four structural genes.

In the brown module, nine structural genes were identified, namely *CsPFKA2* (Cs_ont_2g000110), *CsRPI4* (Cs_ont_2g008890), *CsRER6* (Cs_ont_7g002290), *CsBCAT2* (Cs_ont_8g027050), *CsBCAT6* (Cs_ont_8g027100), *CsAAT1* (Cs_ont_6g013390), *CsAAT3* (Cs_ont_4g003990), *CsTSJT1* (Cs_ont_2g005360), and *CsASNS1* (Cs_ont_8g000470). We constructed a co-expression network using nine structural genes with transcription factors (TFs) in the brown module (**Figure 9**). Forty one highly correlated TFs with at least seven structural genes were identified in the co-expression network, including five *NACs*, four *AP2/ERF-ERF*s, three *MYB*s, two *bHLH*s, two *bZIP*s, two *HB-HD-ZIP*s, two *FAR1*s, two *HSF*s, two *SBP*s, one *WRKY*, one *PLATZ*, one *C2C2->C2C2-Dof1*, one *C2C2*, one *C3H* (**Table S4**). Among these genes, 12 TFs were associated with eight structural genes (R>0.8, P<0.05), including two *MYB*s (Cs_ont_5g004340, Cs_ont_4g005020), two *FAR1s* (Cs_ont_9g001870, novel.674), two *AP2/ERF-ERFs* (Cs_ont_5g036830, Cs_ont_9g013670), one *NAC* (Cs_ont_1g027680), one *GRAS* (Cs_ont_1g023430), one *SBP* (Cs_ont_2g004750), one *EIL* (Cs_ont_3g022790), one *HB-HD-ZIP* (Cs_ont_8g005060), and one *LOB* (Cs_ont_8g027160). Therefore, the nine structural genes and 12 TFs might play an essential role in amino acid biosynthesis in CT and CJ peels. Furthermore, the expression patterns of these 21 genes were analyzed by qRT-PCR (**Figure S7**). The relative expression levels of these genes were basically consistent with FPKM values in RNA-seq, with a few exceptions, indicating the reliability of the transcriptome data overall.

## Discussion

### Amino acid components in CT and CJ peels and the effects of different rootstocks grafting on amino acid biosynthesis

High nutritional content of fruit will drive customers’ desire to buy as consumption concepts and living standards rise. Citrus peel has a wide range of nutrients, including AAs, aroma and flavonoids, which are extremely beneficial to human health (Nair et al., 2018; Matsuo et al., 2019; Sharma et al., 2022a). Studies have shown that the content and type of AAs affect the fruit flavor. And as the fruit ripening, AAs would gradually degrade which provided the basis for the biosynthesis of aromatic substances, thus improving the fruit quality (Gonda et al., 2010; Maoz et al., 2022). The peel and pulp of citrus are rich in AAs, and there are significant differences in amino acid content at different developmental stages. In our preliminary experiment, we found that total amino acid content in the peel was higher than the flesh. A similar phenomenon has been found in quince (Silva et al., 2004). Therefore, the study of AAs in citrus peel could provide relevant information for the development of valuable citrus by-products.

Amino acid biosynthesis is affected by many internal factors and external environment. Previous studies have reported that different varieties, different tissues, heat treatment and nitrogen deficiency affected amino acid biosynthesis (Hanks et al., 2003; Silva et al., 2004; El-Kereamy et al., 2012; Fernández-Fígares et al., 2014; Sun et al., 2017). In this experiment, we found that different rootstock grafts also affected amino acid biosynthesis in citrus peels. After rootstocks grafting, amino acid content was significantly different, and there were also significant differences in different development stages (**Figure 1C**). In the fruit expansion stage, the total amino acid content in the peel reached a peak, but the total amino acid content decreased in the maturity stage. These might be related to the gradual formation of aroma substances with the fruit ripening, and AAs gradually transformed into aroma substances as the basis of metabolite synthesis. However, the underlying reason for the differences and the molecular mechanism which influenced amino acid biosynthesis was unknown. Therefore, metabolomics and transcriptomics analyses of the samples were performed to identify DAAs and molecular mechanisms of amino acid biosynthesis.

Previous studies discovered 38 AAs in different tissues of citrus, such as flesh, peel, leaf and phloem (Matsumoto and Ikoma, 2012; Setamou et al., 2017; Sheng et al., 2017; Hussain et al., 2022). In our experiment, a total of 110 kinds of AA were detected, including 5 essential AAs. A significant number of AAs were uncovered in citrus peel for the first time. Although three essential AAs (L-Isoleucine, L-Leucine and L-Threonine) were not detected, their derivatives were detected (**Table S2**). L-Valine was a branched-chain amino acid that has several uses in food, medicine, and feed (Liu et al., 2021). The content of L-Valine was the highest among the five essential AAs (**Table S2**), which was expected to be used in the food and medical industries. In addition, L-Lysine derivatives (N (6), N(6)-Dimethyl-L-lysine) had the highest content among 110 AAs, which have been found to play an important role in virus control in clinical practice (Pedrazini et al., 2022). The study also found that 10 AAs were affected by rootstock grafting and 11 AAs were affected by development stage (**Figures 2D**, **3**). L-Asparagine was affected not only by development stage, but also by rootstock grafting (**Figure 3**; **Table S2**). Several studies have reported that L-Asparagine, in addition to being a component of protein biosynthesis, played an important role in cancer therapy (Jiang et al., 2021; Halbrook et al., 2022). Therefore, the selection of appropriate rootstock grafting would help to enhance the nutritional value of citrus.

### Gene regulation underlying amino acid accumulation in CT and CJ peels after grafting different rootstocks

Amino acid biosynthesis is subject to strict regulatory mechanisms. PCA and Boxplot results were similar with total amino acid in CT and CJ (**Figures 4A, B**), showing that our results had good repeatability and accuracy. Intracellular signal transduction is mediated by external factors, which influence the synthesis of plant metabolites (Ruan, 2014; Klumpen et al., 2021; Song et al., 2021; Dong et al., 2022). In this study, the KOG functional classification of consensus sequence results revealed most of DEGs might be related to amino acid biosynthesis, including DEGs of signal transduction (**Figure 4D**). GO and KEGG analysis also found significant enrichment of many DEGs in pathways which related to amino acid biosynthesis, such as citrate cycle, photosynthesis, metabolic pathways, and glycolysis (**Figures 5D, E, F**). In addition, these DEGs were associated with developmental stages in **Figure 5D** and **Figure 5E**, while DEGs were associated with rootstock grafting in **Figure 5F**. The GO enrichment terms were inconsistent in CT and CJ peels, which might be the reason for their significant differences in amino acid biosynthesis. The above results showed that there was a significant difference in biological processes and molecular functions in CT and CJ, which might contribute to their different levels of amino acid biosynthesis.

Tricarboxylic acid (TCA) cycle was a major respiratory pathway, and its intermediate products were also involved in the biosynthesis of AAs (D'Este et al., 2018). For example, oxaloacetic acid was a substrate for L-aspartate, L-lysine, and L-asparagine biosynthesis, and alpha-ketoglutaric acid was converted by aspartate aminotransferase (AAT) to glutamate, which could be further converted to L-ornithine and L-arginine (Hu et al., 2022). It was found that the TCA cycle affected the downstream amino acid content in the experiment (**Figure 7**). The change of metabolite content is determined by both structural genes and regulatory genes. An efficient method for analyzing metabolic pathways is to integrate the metabolome and transcriptome (Zhao et al., 2021). RDA has proven to be an effective technique for identifying important factors in metabolite biosynthesis (Wen et al., 2017; Hu et al., 2022). In our study, L-lysine, L-ornithine, and L-arginine were expressed in accordance with their corresponding structural genes (**Figure 9**). In addition, it was found that the expression of *CM* gene promoted the biosynthesis of L-phenylalanine in both CT and CJ, while *PK* and *PFK* were negatively correlated with most amino acid contents in CJ (**Figures 8A, B**). Studies have shown that overexpression of *ASNS* and *PK* positively influenced amino acid content and biosynthesis (Jazmin et al., 2017; Lou et al., 2022). The expression of *PK* and *PFK* genes had greater influence on CJ, which might be the reason for the accumulation of differences of AAs in CT and CJ.

TFs influence metabolite biosynthesis through regulating gene transcription and protein synthesis (Verslues and Sharma, 2010; Lou et al., 2022). In our study, 41 structural gene-related TFs were identified through WGCNA, among which 12 TFs were highly correlated with eight structural genes (**Figures 8**, **9**). Four TFs were MYBs and AP2/ERF-ERF family member of these 12 TFs. Previous studies showed that overexpression of OsMYB55 promoted the total amino acid content and single amino acid content in rice (El-Kereamy et al., 2012). AP2/ERF-ERF might influence amino acid biosynthesis in kiwifruit (Li et al., 2022). Therefore, these TFs might regulate amino acid biosynthesis. However, the effect of these TFs in regulating amino acid biosynthesis remains to be further investigated. These results provide important information for the wider application of citrus peel in the food industry.

## Conclusions

**Figure 10:**
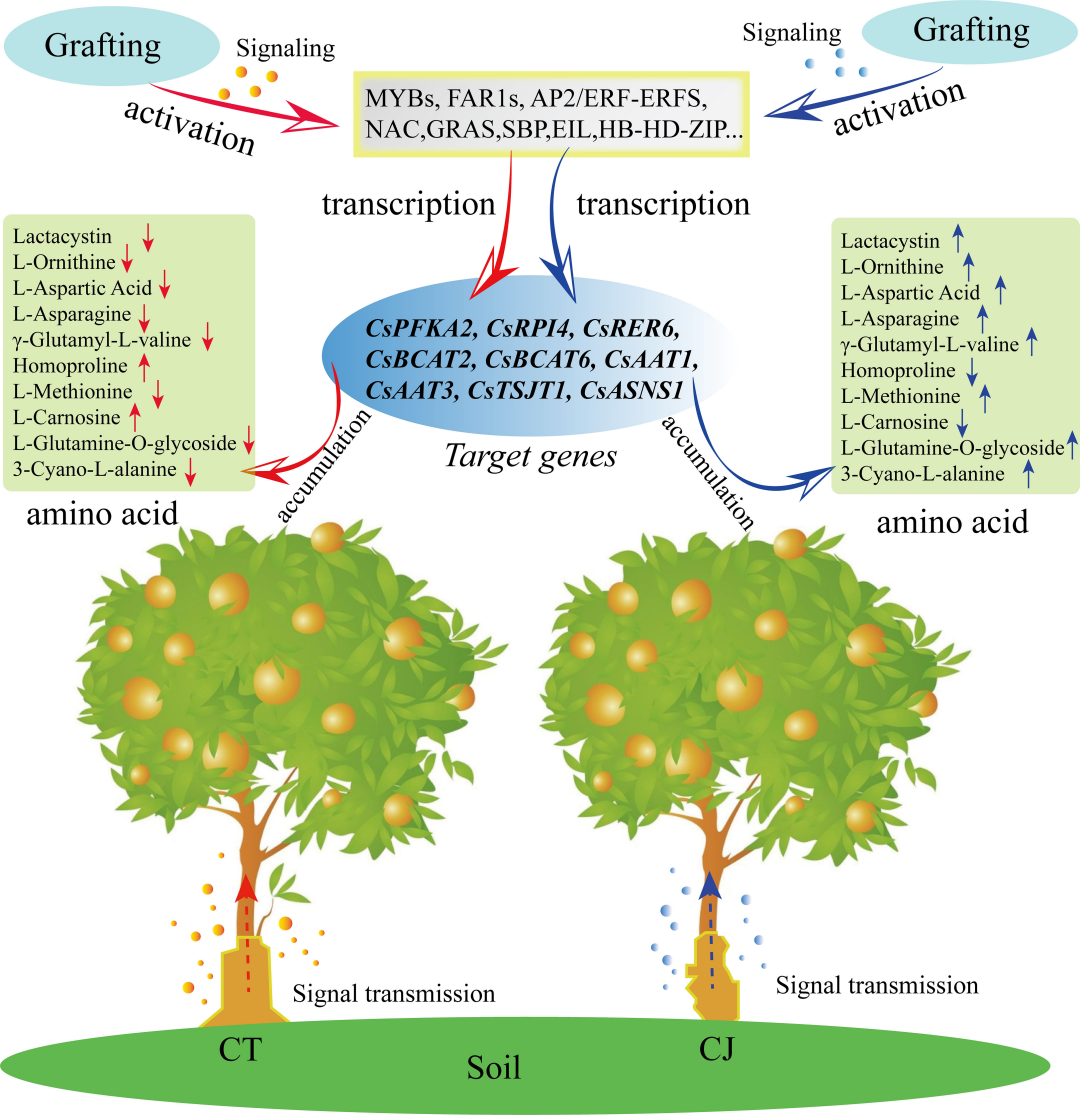
Model diagram of influence of rootstock grafting on AAs differential biosynthesis. The red arrow represents CT. The blue arrow represents CJ. ‘↑’ indicates an increase in amino acid content, and ‘↓’ shows a decrease in amino acid content.

In this study, we conducted a comprehensive analysis of amino acid metabolism and transcriptome to gain insights into the amino acid biosynthesis in CT and CJ peels. Our results indicated that CT and CJ peels contain a total of 110 AAs and their derivatives, including five essential AAs. Moreover, we identified 11 AAs that showed significant correlation with developmental period, and another 10 AAs that were affected by different rootstock grafting. Transcriptome data analysis revealed that 9681 genes were differentially expressed during fruit development. Six hub genes involved in amino acid biosynthesis, including CsCM, CsRPI, CsBCAT, CsSERA, CsMETE, and CsACO, were identified by correlation heatmaps and RDA analysis. In addition, we identified 41 TFs that might involve in amino acid biosynthesis in CT and CJ peels using WGCNA analysis (**Figure 10**). The results provide valuable insights into amino acid biosynthesis in CT and CJ peels and useful information for developing functional citrus food. These findings also have a far-reaching impact on expanding citrus fruit applications in the food industry, making them more attractive to consumers.

## Data availability statement

The original contributions presented in the study are publicly available. This data can be found here: NCBI Sequence Read Archive (BioProject: PRJNA946264).

## Author contributions

Conceptualization, data curation, software, and writing-original draft, BX and QL. Investigation and validation, JY, WZ, and LL. Software, YO and YH. Data curation, GS, SH, and JH. Resources, XW, HD, MZ, and XZ. Funding acquisition and project administration, ZW. All authors contributed to the article and approved the submitted version.
