# Supplementary material for: Transcriptome and UPLC-MS/MS reveal mechanisms of amino acid biosynthesis in sweet orange ‘Newhall’ after different rootstocks grafting
Source: Front Plant Sci. 2023 Jul 11;14:1216826. doi: 10.3389/fpls.2023.1216826 (PMC10366444; doi:10.3389/fpls.2023.1216826)
Supplement: Supplementary file 1 [file DataSheet_1.pdf]

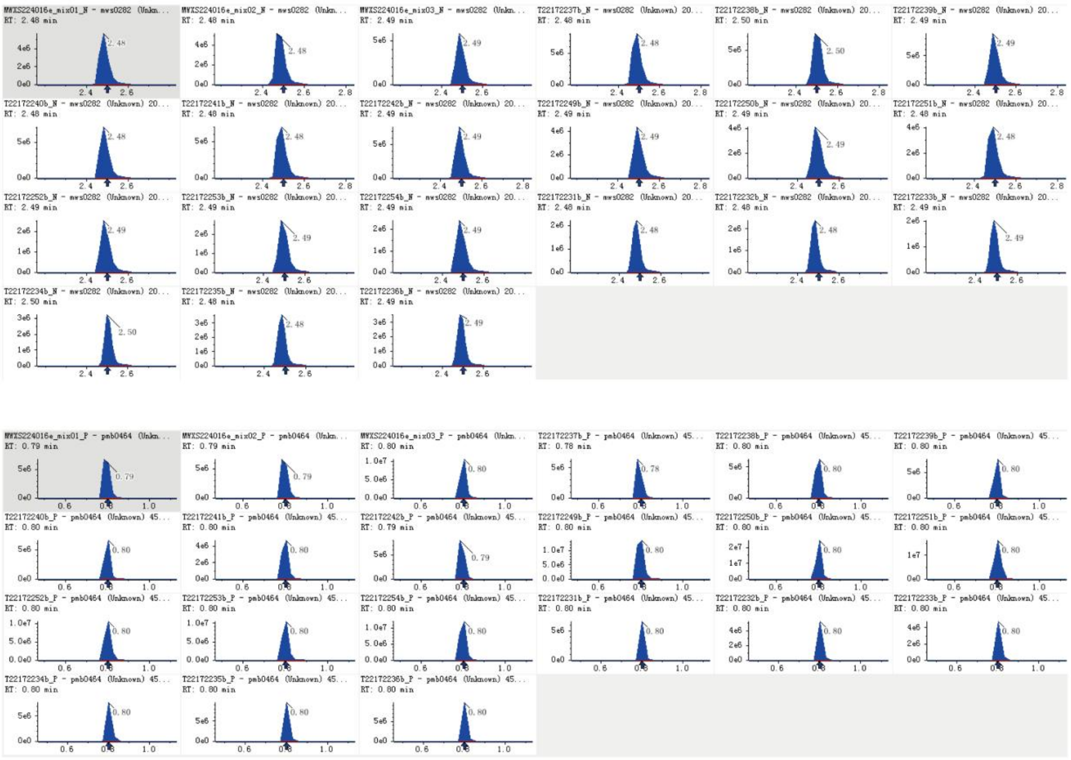

Fig. S1. MRM metabolite detection multi-peak diagrams.

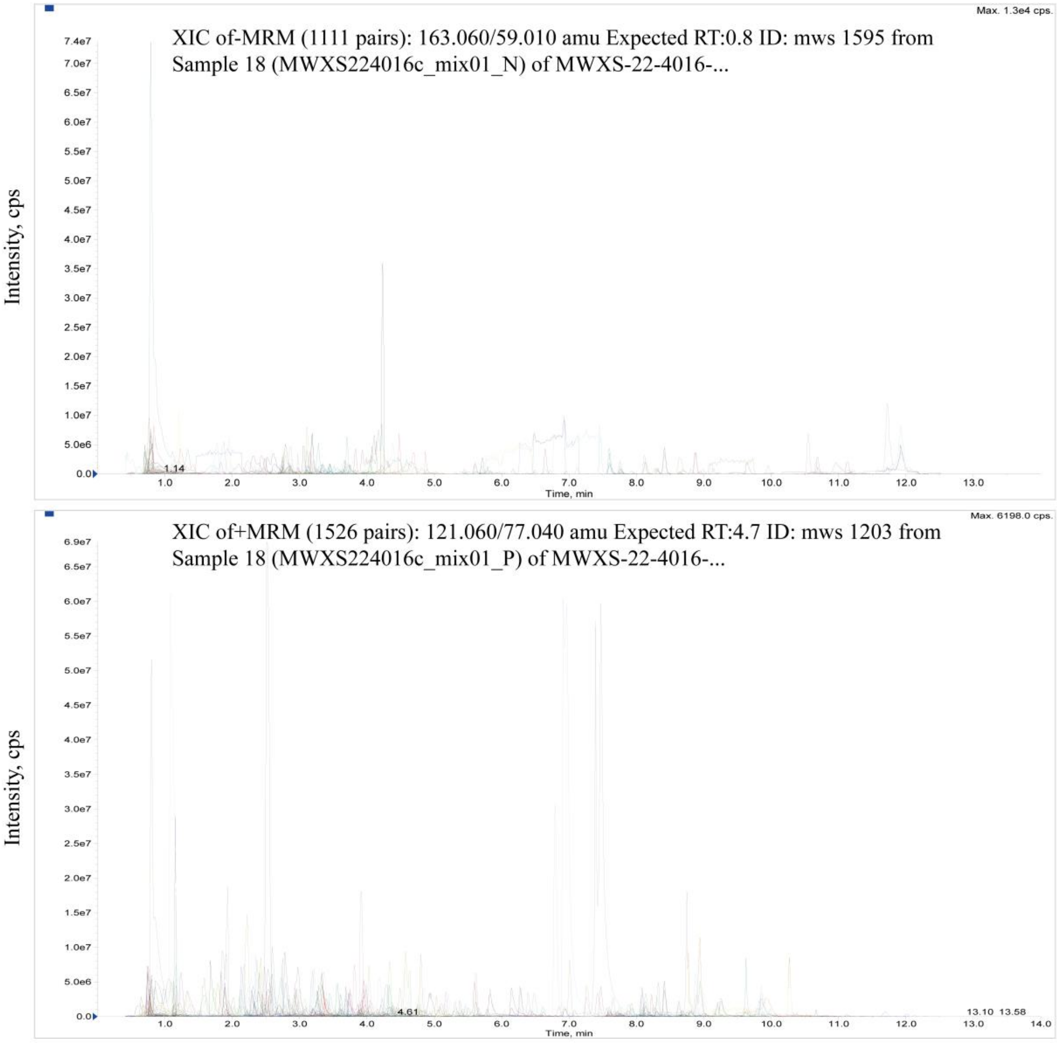

Fig. S2. Multimodal map of multiple reaction monitoring (MRM) metabolite detection. Above was the negative ion mode, below was the positive ion mode. This diagram showed the metabolites that could be detected, with each color-different peak representing one metabolite. The peak area of each chromatographic peak represented the relative content of the metabolite.

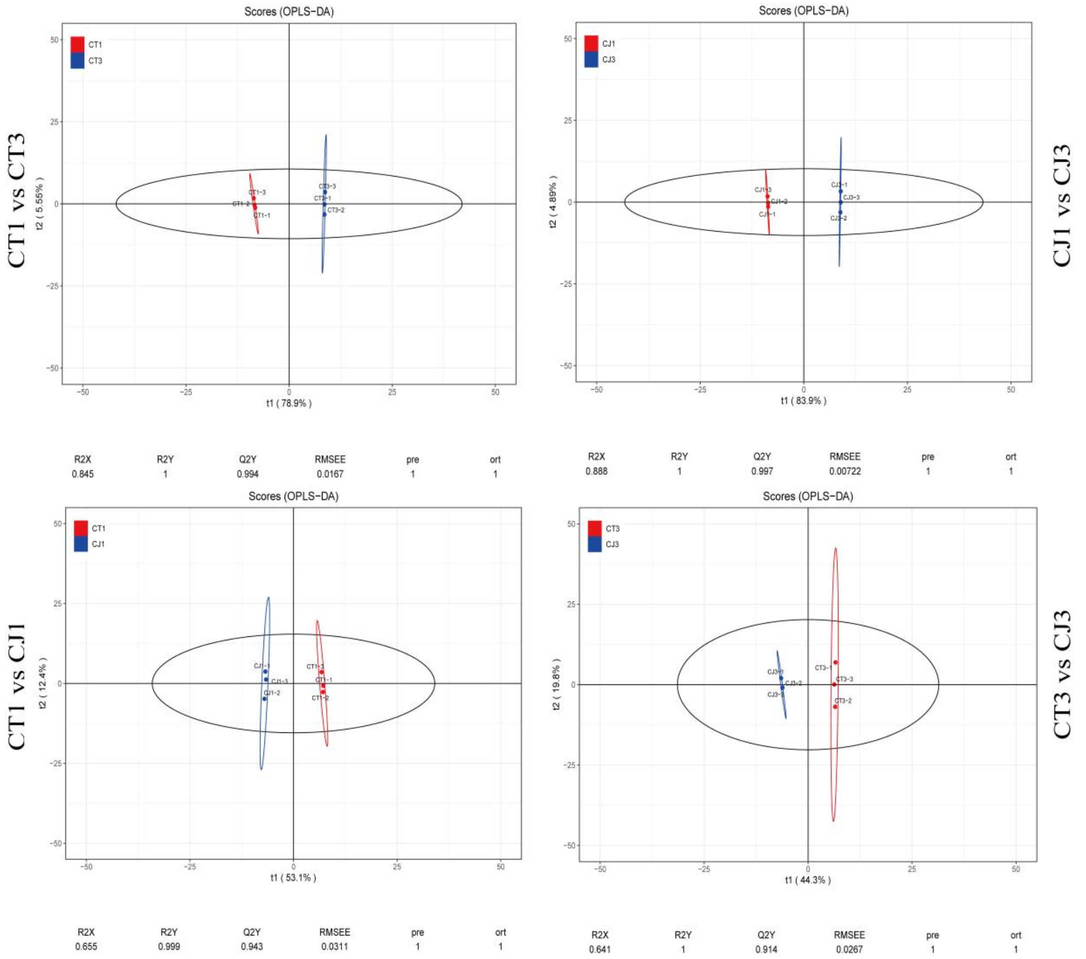

Fig. S3. OPLS-DA scores in CT1 vs. CT3, CJ1 vs. CJ3, CT1 vs. CT3, and CJ1 vs. CJ3, respectively.

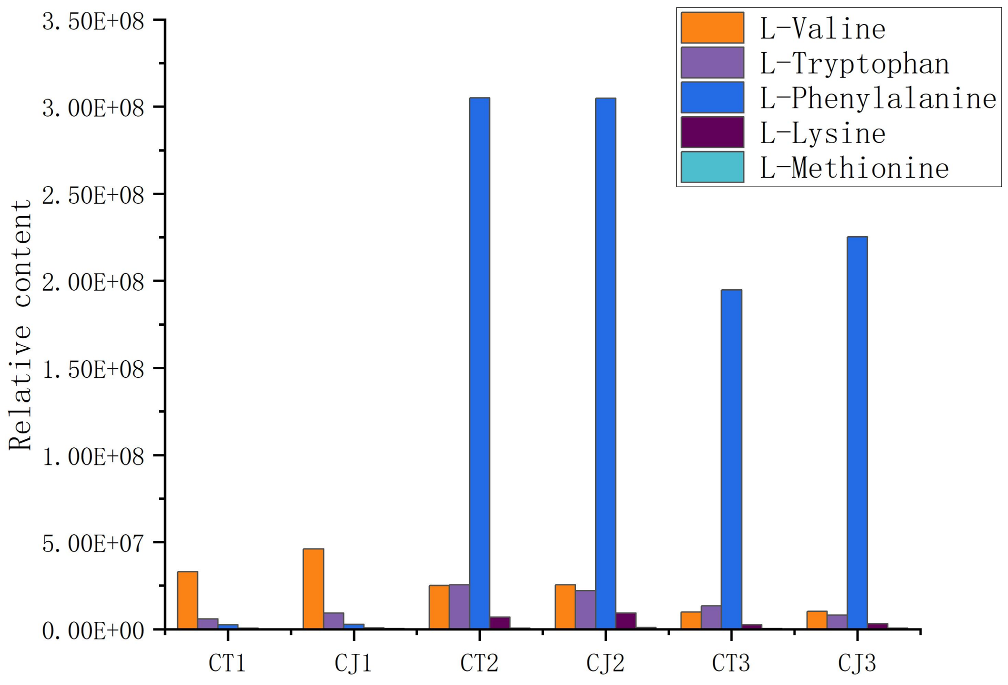

Fig. S4. Relative contents of 5 essential amino acids in 90DAF, 210DAF, and 240DAF.

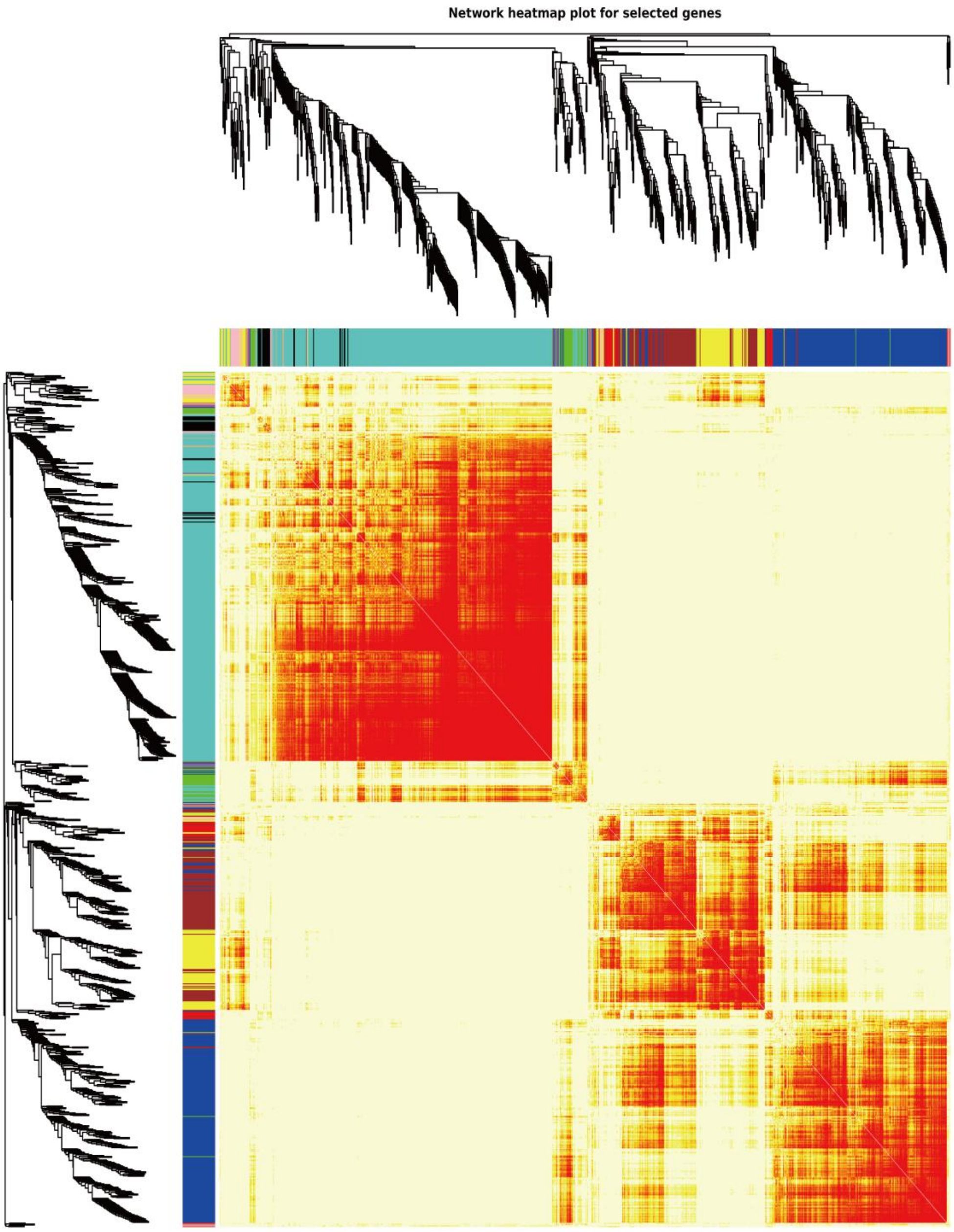

Fig. S5. Module gene clustering heatmap. A topology overlap heatmap is drawn by randomly selecting 1000 genes. Each dendrogram represents a module, each branch represents a gene, and the color of each point represents the strength of the connectivity between the two genes corresponding to the row and column, with darker colors (white, yellow and red) indicating stronger connectivity.

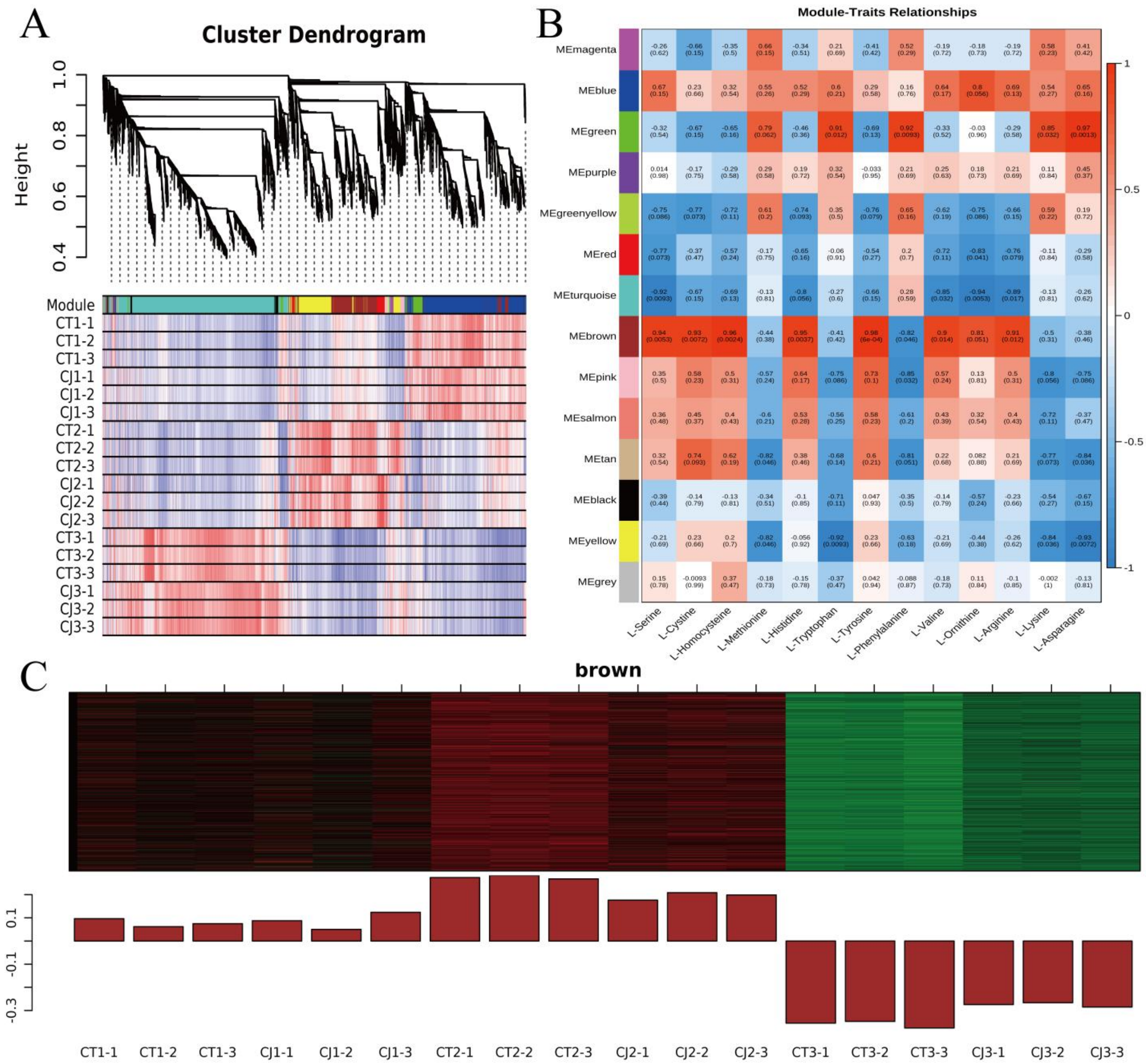

Fig. S6. Weighted gene co-expression network analysis of DEGs. (A) Cluster dendrogram of all DEGs with 18 samples. (B) Module-trait relationship heatmap of 13 modules and 13 AAs. (C) Gene expression heatmap of the brown module. The below column diagram represents the total variation trend of genes.

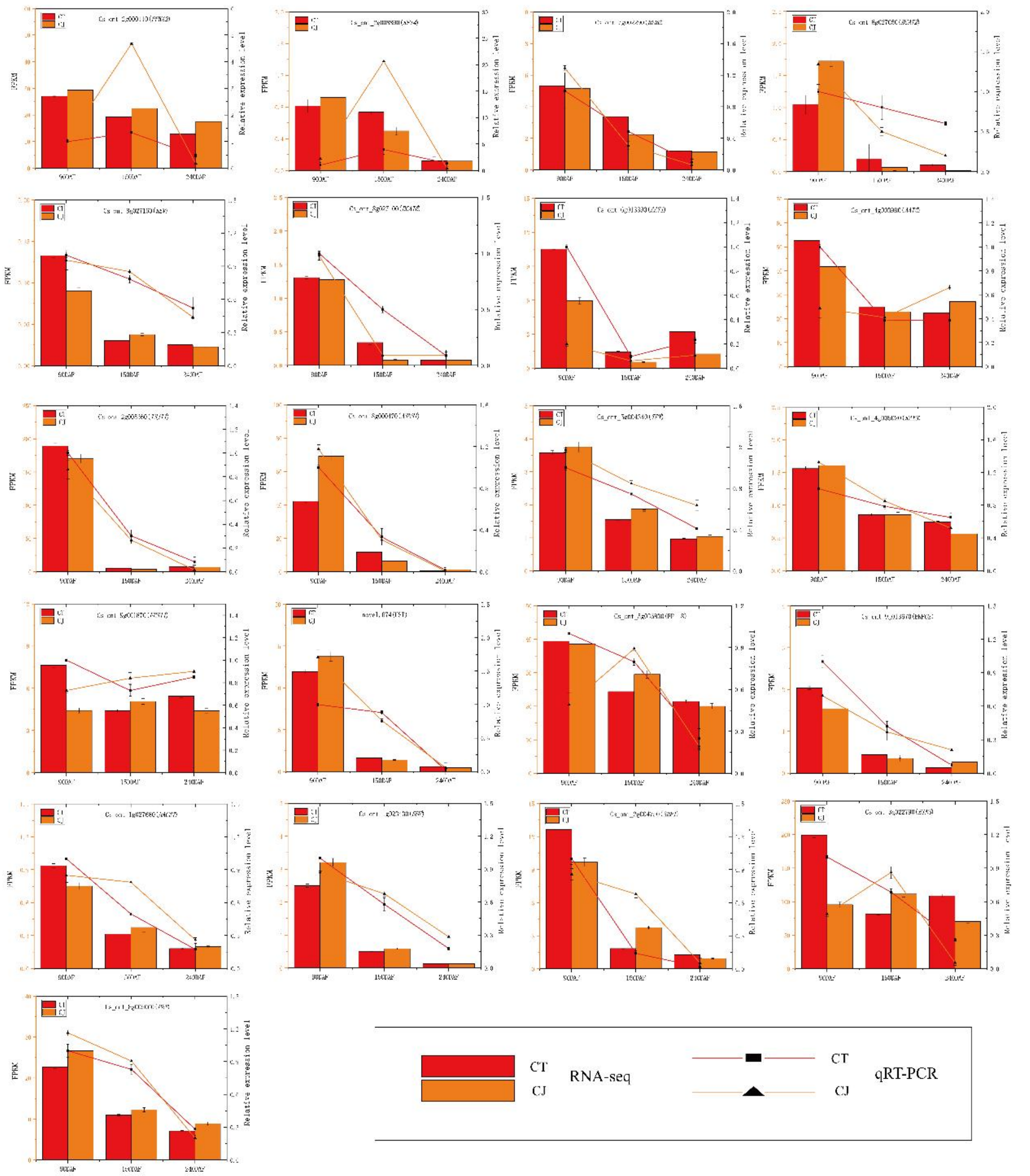

Fig. S7. qRT-PCR validation of 21 genes in amino acid biosynthesis pathway. The column chart shows the FPKM values in RNA-seq data. The line diagram represents the relative expression level in qRT-PCR.
